# Snaring via a Femoral Approach to Facilitate Transvenous Lead Extraction of an Infected Right Ventricular Lead Jailed by a Bioprosthetic Tricuspid Valve

**DOI:** 10.19102/icrm.2024.15105

**Published:** 2024-10-15

**Authors:** Sapan Bhuta, Sena Colak, Aleena I. Arif, Muhammad R. Afzal

**Affiliations:** 1 Electrophysiology Section, Division of Cardiovascular Medicine, The Ohio State University Wexner Medical Center, Columbus, OH, USA

**Keywords:** Jailed lead, laser sheath, snare, transvenous lead extraction, tricuspid valve replacement

## Abstract

An 85-year-old woman presented with *Corynebacterium* bacteremia complicated by infective endocarditis with vegetations on the prosthetic mitral valve and right ventricular (RV) lead. The patient had a single-chamber permanent pacemaker with two RV leads, one of which was previously trapped or “jailed” after a bioprosthetic tricuspid valve replacement. Complete transvenous lead extraction including the chronically retained jailed RV lead was achieved via laser extraction assisted by concomitant traction from a superior left subclavian and inferior right femoral venous approach.

## Introduction

The indications, volume, and longevity of cardiac implantable electronic devices (CIEDs) have dramatically increased over time.^1^ Consequently, the need for lead extraction has also risen, with common indications including infection, lead malfunction, device upgrade, tricuspid regurgitation, and venous obstruction.^2^ However, transvenous lead extraction (TLE) is a technically challenging procedure, particularly in patients with chronic retained leads, with a high risk of serious complications such as lead breakage, venous or myocardial tear, tamponade, or death.^3,4^ Prior tricuspid valve replacement (TVR), resulting in the trapping of leads between the native tricuspid valve annulus and the outer ring of the prosthetic valve, is a contraindication for TLE.^5^ However, failure to completely extract the infected leads can result in significant morbidity and mortality.^1^ Here, we report a case of a TLE of a right ventricular (RV) lead jailed by a bioprosthetic tricuspid valve accomplished using a combined subclavian and femoral approach.

## Case presentation

An 85-year-old woman with a history of permanent atrial fibrillation, single-chamber pacemaker implantation via a left prepectoral approach 11 years ago, and a bioprosthetic TVR and bioprosthetic mitral valve replacement 8 years ago was transferred to The Ohio State University Wexner Medical Center for infective endocarditis. Her past medical and surgical history was notable for a bioprosthetic TVR, during which the RV lead was jailed by the prosthetic valve. Initially, the RV lead continued to be functional, but, due to a malfunction of the jailed RV lead, a new transvenous RV lead was subsequently implanted 4 years ago through the prosthetic valve.

The patient initially presented to an outside hospital emergency department for generalized weakness and a fall at home. She complained of pain secondary to the fall, fevers and chills, and poor oral intake. The patient was noted to be ill-appearing and very lethargic, which was secondary to toxic metabolic encephalopathy in the setting of dehydration, hyponatremia, and occult sepsis. Her vitals were unremarkable except for a heart rate of 150 bpm. An electrocardiogram was consistent with atrial fibrillation with a rapid ventricular response, while the following laboratory results were notable: sodium, 125 mEq/L; high-sensitive troponin, 68.50 ng/L; procalcitonin, 1.88 ng/mL; erythrocyte sedimentation rate, 60 mm/h; and C-reactive protein, 21.1 mg/dL. The patient was empirically started on ceftriaxone and admitted. Subsequently, a set of blood cultures were confirmed positive for *Corynebacterium striatum*. Antibiotics were escalated to vancomycin based on sensitivities. An abdominal ultrasound revealed a distended gallbladder with sludge and thickening concerning for cholecystitis, and the patient underwent a robotic cholecystectomy.

**Figure 1:**
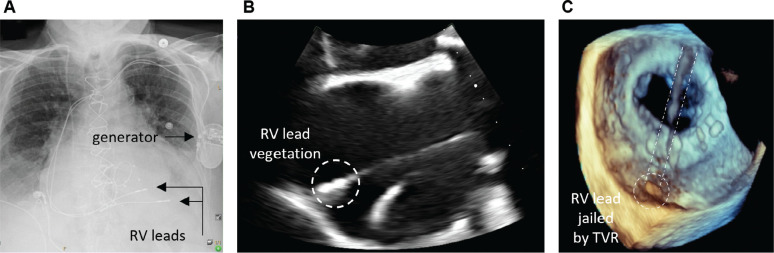
**A:** A postero-anterior chest X-ray demonstrating a permanent pacemaker generator with two right ventricular (RV) leads, one previously abandoned and the other jailed after a bioprosthetic tricuspid valve replacement. **B:** A two-dimensional transesophageal echocardiogram demonstrating an RV lead vegetation. **C:** A three-dimensional transesophageal echocardiogram demonstrating the RV lead entrapped “jailed” by the bioprosthetic tricuspid valve. *Abbreviations:* RV, right ventricle/ventricular; TVR, tricuspid valve replacement.

Postoperatively, the patient continued to have persistent bacteremia despite intravenous vancomycin therapy. Infective endocarditis was suspected, as the identified species is known to be associated with indwelling catheters and hardware infections. A transthoracic echocardiogram was unremarkable with a preserved left ventricular ejection fraction of 55% and normally functioning prosthetic valves. A transesophageal echocardiogram demonstrated a small, echo-dense structure at the tip of the RV lead suggestive of a vegetation **(Figures 1A–1C)** and a mobile vegetation on the atrial side of the mitral valve ring measuring 0.6 cm. To address the persistent bacteremia and endocarditis, the decision was made to transfer the patient to a high-volume lead extraction center to pursue a high-risk extraction.

**Figure 2:**
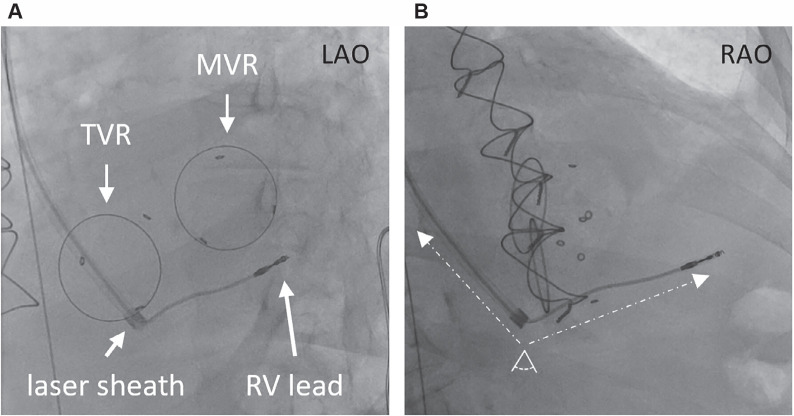
Left **(A)** and right **(B)** anterior oblique projections where the laser sheath is advanced proximal to the ring of the bioprosthetic tricuspid valve. Despite adequate traction on the lead from the subclavian approach, the lead tip could not be freed possibly due to the presence of an acute angle between the portions of the lead outside and within the right ventricle. *Abbreviations:* LAO, left anterior oblique; MVR, mitral valve replacement; RAO, right anterior oblique; RV, right ventricle/ventricular; TVR, tricuspid valve replacement.

**Figure 3:**
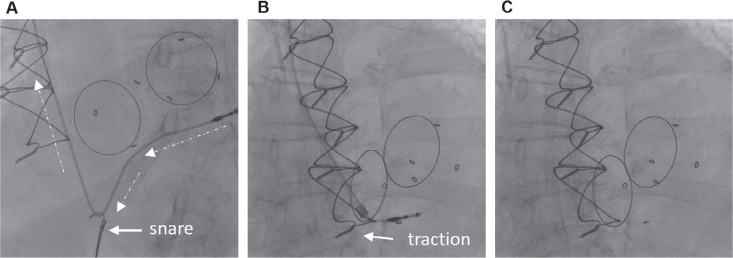
**A and B:** Use of a tip-deflecting wire via a femoral approach to exert co-axial traction on the lead tip resulted in detachment of the lead from the ventricular wall. After the lead detached from the ventricular wall, traction from the subclavian approach resulted in the complete removal of the lead. **C:** No remnant of the lead was noted after lead extraction.

The generator pocket was opened using blunt dissection, electrocautery, and sharp dissection. There was no evidence of purulence in the pocket. The generator was carefully dissected free of the fibrous tissues, with close attention paid to avoid causing damage to the lead system. The generator (Accent SR Model PM1110; Abbott, Chicago, IL, USA) was removed from the pocket, and hemostasis was then obtained using electrocautery. Using the standard stylets, the helix on both leads was retracted. The RV leads were then prepped with a lead locking device (LLD EZ; Philips, Andover, MA, USA). An extraction of the more recent lead (Tendril STS Model 2088TC; Abbott) at the RV apex was attempted using a superior approach through the left subclavian vein. The lead was successfully extracted with gentle traction. An extraction of the abandoned lead (Tendril SDX Model 1688TC; Abbott) at the RV apex was then attempted using a superior approach through the left subclavian vein. A 14-French (Fr) laser sheath (GlideLight; Philips) was used to free up the lead in the innominate vein and superior vena cava. The laser sheath was advanced into the right atrium close to the annulus of the TVR **(Figures 2A and 2B)**. The laser sheath was not advanced further to avoid damage to the annulus of the tricuspid valve. Despite traction, the lead could not be freed. A 16-Fr sheath (Introducer Sheath Set Femoral 16 Fr; Cook Medical, Bloomington, IN, USA) was then advanced into the right femoral vein. Through the 16-Fr venous access, a deflectable guidewire (Reuter Tip-Deflecting Wire Guide; Cook Medical) and a 25-mm snare (Amplatz Goose Neck Vascular Snare Kit; Medtronic, Minneapolis, MN, USA) were advanced. The deflectable guidewire was advanced over the lead. Traction was applied superiorly, and counter-traction was applied inferiorly, translating into co-axial traction on the lead that resulted in detachment from the ventricular septum. At this point, a low-profile 12-Fr laser sheath (GlideLight; Philips) was advanced over the lead from a left prepectoral approach. Continued gentle traction on the lead resulted in complete removal of the lead **(Figures 3A–3C)**. There was no hemodynamic compromise at the end of the procedure. Transesophageal echocardiography was performed during the procedure. Following extraction of the entrapped lead, there was no evidence of a paravalvular leak or worsening tricuspid regurgitation. The pocket was closed with primary intention. The 16-Fr femoral venous sheath was removed, and a modified figure-of-eight stitch using a stopcock was applied.

The patient recovered well postoperatively and was discharged to a skilled nursing facility to allow completion of a 6-week course of vancomycin. Notably, intraoperative device cultures also grew *C. striatum*. After 6 weeks, the patient was seen in an infectious disease clinic and noted to be feeling well and in good condition. The patient was placed on indefinite suppression with doxycycline given concomitant prosthetic valve endocarditis.

Consent was obtained from the patient for the publication of this manuscript.

## Discussion

This case report describes the experience of transvenous extraction of leads jailed by a bioprosthetic tricuspid valve. This is particularly important for patients with infective endocarditis, lead-related vegetations, and persistent bacteremia. There are a few perceived challenges while attempting to extract a lead jailed by a prosthetic valve. First, the lead is compressed between the ring of the prosthetic valve and the annulus of the native valve. When a mechanical tool such as a laser or powered sheath is advanced over the lead, there is a potential risk of cardiac perforation that may require a sternotomy for open repair. Due to a previous history of cardiac surgery, such patients often have a prohibitive risk for reoperation. Second, potential damage to the ring of the prosthetic valve can lead to significant paravalvular leak, valvular regurgitation, or valve dehiscence. Overall, we believe that the risk and benefits of the extraction of jailed leads should be meticulously assessed prior to the procedure. In this case, the risk of persistent bacteremia and infective endocarditis was felt to be high; therefore, a complete system extraction was contemplated.

The contemporary practice is to abandon CIED extraction in patients with any lead jailed by a prosthetic valve. A complete system extraction can be attempted despite the presence of jailed leads in patients in whom resolution of infection is unlikely without lead extraction.^6^ It should be noted that unique patient and lead characteristics coupled with operator experience with lead-extraction procedures could have led to a successful outcome in this unique and challenging situation. This situation may not be widely applicable, and the extraction of an entrapped lead is a challenging situation that can result in catastrophic complications requiring an emergent sternotomy; therefore, a shared decision-making conversation regarding the elevated risks and potential benefits should precede such procedures.

Lastly, surgical repair of tricuspid valve regurgitation in the presence of transvenous leads is a unique situation. Due to significant risks associated with the extraction of a jailed/entrapped lead, it is always recommended to perform lead extraction prior to prosthetic TVR. To minimize the need for repeat device implantation via a transvenous route, epicardial leads can be placed during valve repair. Epicardial lead placement also avoids the risk of damage to the freshly implanted prosthetic valve from transvenous leads. With the advent of newer tricuspid valve repair strategies, such as transcatheter edge-to-edge repair (TEER), it is conceivable that valve repair can be performed in the presence of transvenous leads but still avoiding the risk of lead entrapment. The clips used during TEER can be applied in a way where the lead is not entrapped between the clip and the tricuspid annulus.

## Conclusion

TLE of leads jailed by a prosthetic tricuspid valve can be attempted in very select situations. TLE of such leads can be attempted in cases with a high risk of persistent bacteremia and infective endocarditis. Powered sheaths should not be used through the tricuspid valvular annulus; rather, co-axial traction can be attempted using superior and inferior approaches to facilitate extraction.
